# The Inference of Friendly Communicative Atmosphere Created by Geometric Shapes

**DOI:** 10.1177/2041669517744571

**Published:** 2017-12-11

**Authors:** Masahide Yuasa

**Affiliations:** Department of Applied Computer Science, 84131 Shonan Institute of Technology, Fujisawa, Kanagawa, Japan

**Keywords:** atmosphere, geometric shape, friendliness, interaction, synchronization, coincidence

## Abstract

Many previous studies on inference of social behaviors using geometric shapes have explored causality, animacy, intention, and desire inferred from the movements of such shapes; however, inference of communicative atmosphere in terms of friendliness/antagonism using geometric shapes has not yet been studied well. This study investigated how a friendly/antagonistic communicative atmosphere was inferred from the movement of two egg shapes. We developed animations for these shapes involving forward/backward/parallel tilts with coincidence/incoincidence of synchronous movement. Results showed significant differences in the inference of friendly/antagonistic atmosphere between coincident and incoincident synchronous movement. In addition, the inference of a friendly atmosphere was affected by the combination of forward tilt with incoincident movement, which may be interpreted as interaction between the shapes, such as responding or providing feedback. This suggests that individuals may infer a friendly/antagonistic communicative atmosphere from both coincident movement and incoincident movement interpreted as an interaction.

## Introduction

When children play with dolls, they can pretend that dolls are talking to one another by having them lean forward towards each other, even if the joints in their arms and legs do not move. Furthermore, using the angle or timing of the tilt, children can show that the dolls are getting along or fighting. This action is not just limited to dolls; children can also personify simple objects, such as spoons and pencils, and create a friendly/antagonistic atmosphere between them. Therefore, it is believed that humans have a special capacity to both express and comprehend a friendly/antagonistic atmosphere, even if this atmosphere is created by geometric shapes.

Many previous studies have used geometric shapes to explore the social responses, such as causality, animacy, intention, and desires, that can be inferred by individuals from the movements of such shapes (Gao & Scholl, 2011; Heider & Simmel, 1944; McAleer & Love, 2013; Michotte, 1963; Scholl & Tremoulet, 2000; Tremoulet & Feldman, 2000); however, inferring a friendly/antagonistic atmosphere using geometric shapes has not yet been studied well. Zumthor (2006) and Böhme (1993, 1995, 2005) conducted theoretical analyses of atmosphere in terms of physical materials (i.e., spaces or locations among buildings or objects). However, it is still unknown how individuals infer friendly/antagonistic communicative atmospheres and what particular elements are used in this inference. Our study aimed to deepen our understanding of atmosphere perception and reveal the mechanism of inference of friendly/antagonistic atmosphere using geometric shapes.

Previous studies have mainly focused on trajectory to examine the inference of causality, animacy, and so on. In one of the earliest of such studies, Heider and Simmel (1944) revealed that humans can interpret social relationships based on the trajectories of abstract geometric shapes. McAleer and Love (2013) extracted the trajectory from the actual movement (chasing, fighting, following, etc.) of two persons using computer vision and investigated individuals’ inferences from the extracted trajectories applied to two geometric shapes. Kuhlmeier, Wynn, and Bloom (2004) studied the social perception of helping and hindering behavior using geometric shapes such as triangles, squares, and circles representing eyes and a nose. Gao and Scholl (2011) examined the inference of chasing behavior in terms of how one of two circles approached the other. Gao, McCarthy, and Scholl (2010) and Scholl and Gao (2013) studied what they named the “wolfpack effect,” which refers to how the trajectory and orientation of darts aiming continually toward a target were interpreted as chasing behavior. However, trajectory was not considered relevant for the study of friendly/antagonistic atmosphere, as suggested by children expressing a friendly atmosphere by having dolls tilt toward one another while remaining in the same positions. Therefore, the novelty of the current study lies in examining the expressions of shapes while their positions remain the same.

Furthermore, this study focused on the observation of synchronous movement of geometric shapes to infer a friendly/antagonistic atmosphere. Previous studies reported that synchronization of body postures in a conversation raised affinity between participants and enhanced feelings of empathy and bonding towards each other (Bates, 1975; Chartrand & Bargh, 1999; LaFrance & Broadbent, 1976; Nagaoka, Komori, & Yoshikawa, 2007; Stel & Vonk, 2010). These past studies considered how individuals perceive friendliness in their experience, for instance, as participants in a conversation; however, no research has been conducted on how friendliness can be inferred from the observation of synchronous movements or using geometric shapes. We hypothesized that synchronous movement will influence individuals’ inference of friendly/antagonistic atmosphere and designed coincident and incoincident movements of the geometric shapes to investigate this influence.

## Method

### Design of Synchronization of Geometric Shapes

**Figure 1.**
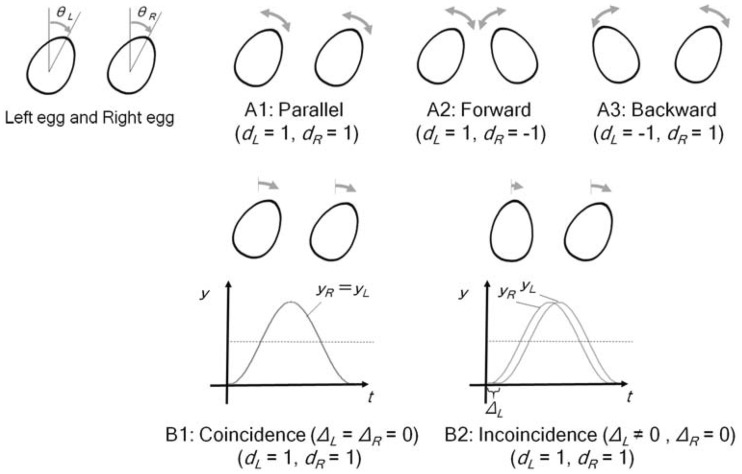
Equations (1) and (2) describe the angle of tilt (*θ_L_* and *θ_R_*) and the reciprocal motions.

In this study, an egg shape was used as a typical geometric shape that can represent the presence of a person. We referred to a Matryoshka doll (Russian nesting doll) and a Daruma doll (Japanese traditional doll), which are very primitive, almost egg-shaped objects with no protrusions that are identified with a person. We created animations of two egg shapes arranged close together (left and right egg in Figure 1). We used synchronous sine functions, which are traditional methods to create synchronous reciprocal motion (Pikovsky, Rosenblum, & Kurths, 2003). We set sine function for both the left and right eggs individually to develop the synchronous movement of the two egg shapes. The following equations (1) and (2) were defined to make such motions.

(1) $$y_{n}(t)=A\text{sin}\{\frac{2\pi }{T}(t-\Delta_{n})-\frac{\pi }{2}\}+\phi$$

(2) $$\theta_{n}(t)=\theta_{\text{max}}d_{n}y_{n}(t)$$

Here, *n* indicates the left or right egg (L or R). Using equations (1) and (2) for the left and right eggs, the eggs move individually but they are synchronized. The *y_n_* in equation (1) is the displacement and the *θ_n_* in equation (2) shows the angle of tilt for each time (*t*). The amplitude (*A*), period (*T*), and nonzero center amplitude (*ϕ*) are constant values and equal for the left and right eggs. The changing parameters (*d_n_* and Δ*_n_*) can create different types of movement for the left and right eggs. Figure 1 explains the movements by changing parameters. The *d_n_* indicates direction: If the *d* value is positive, the egg rotates clockwise, and if the *d* value is negative, it rotates counterclockwise. We can create a perfect synchronous movement of the two eggs (Δ*_L_* = Δ*_R_*) or use different phases for the left and right eggs (Δ*_L_* ≠ Δ*_R_*). In our study, we focused on three types of tilts by varying *d_L_* and *d_R_*. The first one is a parallel tilt in which eggs sway from side to side together, such as in dancing or singing together; the second tilt involves leaning forward (i.e., towards each other), such as in greeting or talking to each other; and the third tilt involves leaning backward (i.e., away from each other), such as in fighting or disliking. Furthermore, we used delay (Δ*_n_*), wherein one egg starts to move some time after the first egg starts moving, to create coincidence (Δ*_L_* = Δ*_R_*) or incoincidence (Δ*_L_* ≠ Δ*_R_*) in the starting time of the movement of the eggs. Therefore, we prepared six animations combining these two factors;

Factor A: tilt

A1: Parallel: leaning in the same direction (*d_L_* = 1, *d_R_* = 1)

A2: Forward: leaning forward towards each other (*d_L_* = 1, *d_R_* = −1)

A3: Backward: leaning backward towards each other (*d_L_* = −1, *d_R_* = 1)

Factor B: timing

B1: Coincidence (Δ*_L_* =Δ*_R_* = 0)

B2: Incoincidence (Δ*_L_* = 1, Δ*_R_* = 0)

In the experiment, we used *θ_MAX_* = 30°, *T* = 3, *A* = 1/2, *ϕ* = 1/2, and Δ*_L_* = 1. We determined these parameters by conducting preliminary experiments. We interviewed a few volunteers to determine whether the movement of eggs was abnormal and meaningless while repeatedly changing these parameters before we finally decided on them. Since the parameters in this experiment are tentative, it is possible that more suitable parameters exist. In the future, we should investigate them through additional experiments.

### Research Hypotheses

We hypothesized that when the eggs lean forward, the communicative atmosphere will be inferred as friendlier than for the other tilt conditions. Price, Peterson, and Harmon-Jones (2011) and Harmon-Jones, Gable, and Price (2011) reported that leaning forward was associated with a higher approach motivation during goal acquisition, such as leaning towards an appetitive dessert or an interesting situation. Forward body tilts may express an individual’s motivation and attitude to communicate with a partner, and this expression might suggest a friendlier communicative atmosphere. On the other hand, leaning backward may express a lack of interest or no motivation to communicate with others, and this expression might be associated with a person who turns his or her eyes or face away when he or she does not want to communicate (Argyle, 1988). Thus, backward body tilts might suggest an antagonistic communicative atmosphere.

Regarding timing, we hypothesized that in the case of forward and parallel tilts, a coincident movement will suggest a friendlier relationship than an incoincident one. Coincident activities, such as synchronized swimming or cheerleading, require a friendly enough relationship to create a harmonious combination and perfectly matching movements. We predict that the coincident patterns of parallel and forward tilts will suggest such coincident activities and friendly relationships; thus, they will be interpreted as friendlier than will incoincident ones. On the other hand, we predict that an antagonistic communicative atmosphere will be inferred from a backward coincident tilt. This is because the meaning of the backward tilt—namely, an aversion to and disinterest in others—might be enhanced by simultaneous movement, suggesting a stronger dislike for each other. A backward incoincident tilt might suggest that only one of the two dislikes the other because of nonsimultaneousness; this might not be worse than the both of them disliking each other.

Based on the earlier discussion, we formulated the following hypotheses:

Hypothesis 1 (influence of coincidence/incoincidence): There is a significant difference between the atmospheres suggested by coincident and incoincident movements.

1. Participants will rate the communicative atmosphere for coincident movement as friendlier than for incoincident movement in the case of parallel and forward tilts.
2. Participants will rate the communicative atmosphere for coincident movement as more antagonistic than for incoincident movement in the case of backward tilt.
  Hypothesis 2 (differences between parallel/forward/backward tilts): There is a significant difference between the atmospheres suggested by parallel, forward, and backward tilts. Participants will rate the communicative atmosphere of the forward tilt as friendlier than that of the backward and parallel tilts.

### Participants and Experimental Procedure

Participants were 100 Japanese university students (50 men and 50 women, mean age = 21.1 years). We asked a research company to recruit participants and to conduct the experiment online. The important issues (e.g., personal information protection) in the experiment were explained to the participants and their consents were obtained before the experiment. The six animations were shown in a random order using web pages; one animation was displayed on each web page. The animation was located in the upper part and the items (questions) were shown in the lower part of each web page. After the participant watched one animation, he or she answered the questions. After finishing this, the next page was shown and the new animation and questions were displayed. The participants could rate each condition only once and could not return to the previous page. Each animation of reciprocal movements lasted for about 20 seconds. The size of the animation was 320 × 240 dots and it was sufficient to answer the questions. Participants were requested to rate each animation on a 5-point scale ranging from 0 to 4 after watching it. We instructed the participants to see “eggs” as figures that communicate each other. We gave the participants the following instructions: “Animations show a scene of two geometric figures communicating with each other. Please rate the scene on a scale of 0–4.” We used two items. Item 1 suggested “I think that these objects portray an antagonistic–friendly atmosphere” (0 = *antagonistic*, 1 = *somewhat antagonistic*, 2 = *neither*, 3 = *somewhat friendly*, 4 = *friendly*) and Item 2 read “I think that these objects’ movements are mechanical–biological” (0 = *mechanical*, 1 = *somewhat mechanical*, 2 = *neither*, 3 = *somewhat biological*, 4 = *biological*). All the data were collected on the web server. We conducted a two-way repeated-measure analysis of variance (ANOVA) for each item (Factor A: tilt and Factor B: timing).

## Results

**Figure 2.**
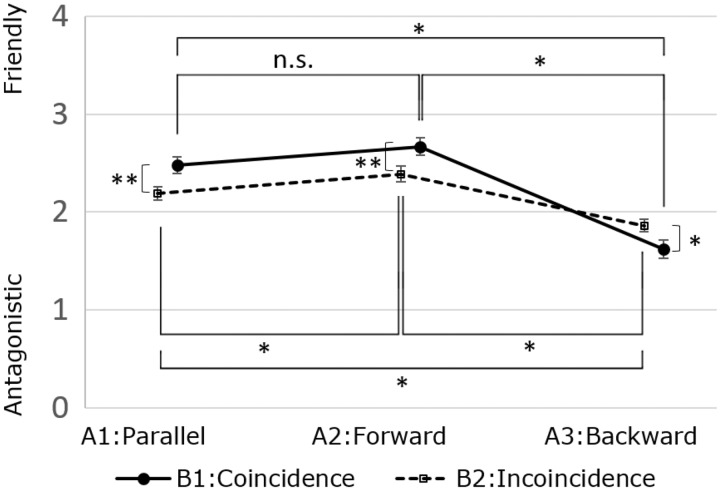
Two-way repeated-measure ANOVA and multiple comparisons using the LSD method for Item 1: friendly–antagonistic atmosphere (**p < .01, *p < .05). Error bars represent standard errors.

**Figure 3.**
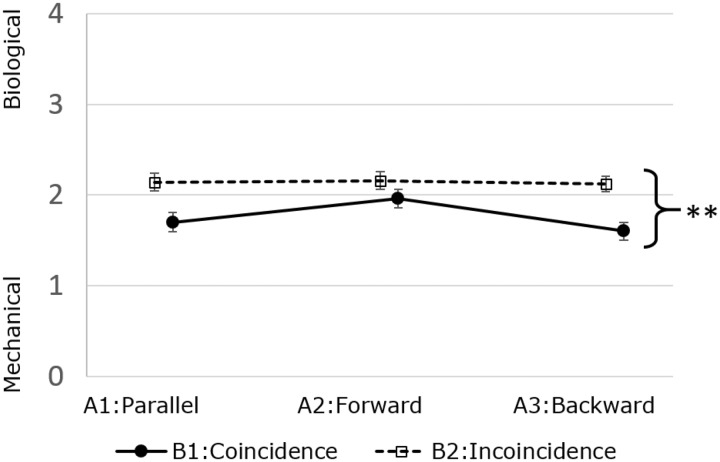
Two-way repeated-measure ANOVA for Item 2: mechanical–biological. **p < .01. Error bars represent standard errors.

Figures 2 and 3 show the average responses for the two items and results of the ANOVAs. Regarding friendliness, there was a significant interaction effect between tilt (A) and timing (B), *F*(2, 198) = 8.82, *p* < .01, *η*^2 ^= .02. A simple main effect was observed for the tilt factor on the friendliness rating both in the coincidence condition (B1), *F*(2, 198) = 41.86, *p* < .01, *η*^2 ^= .22, and in the incoincidence condition (B2), *F*(2, 198) = 15.03, *p* < .01, *η*^2 ^= .08, of the timing factor. A simple main effect of timing on the friendliness rating was also observed for each of the levels of the tilt factor: coincidence (B1) > incoincidence (B2) for parallel tilt (A1), *F*(1, 99) = 8.80, *p* < .01, *η*^2 ^= .03; coincidence (B1) > incoincidence (B2) for forward tilt (A2), *F*(1, 99) = 8.61, *p* < .01, *η*^2 ^= .03; and coincidence (B1) < incoincidence (B2) for the backward tilt (A3), *F*(1, 99) = 4.99, *p* < .05, *η*^2 ^= .02.

In the multiple comparisons for Factor A using the LSD method, in the coincidence condition (B1) of timing, parallel (A1) = forward (A2) > backward (A3) (mean square error [MSE] = 0.75, *p* < .05) and there was no significant difference between parallel (A1) and forward (A2). In contrast, in the incoincidence condition (B2) of timing, there were significant differences as follows: parallel (A1) < forward (A2), parallel (A1) > backward (A3), and forward (A2) > backward (A3;MSE = 0.70, *p* < .05).

Regarding the mechanical–biological rating, there was no significant interaction effect between tilt and timing, *F*(2, 198) = 2.27, n.s., *η*^2 ^< .01. A main effect was observed for timing, *F*(1, 99) = 20.20, *p* < .01, *η*^2 ^= .04. There was a marginally significant main effect of tilt, *F*(2, 198) = 2.59, *p* < .10, *η*^2 ^< .01.

## Discussion

There were significant differences between coincident and incoincident movements. In the case of forward and parallel tilts, coincident movement was rated as portraying a friendlier atmosphere than that portrayed by incoincident movement. In the case of backward tilt, coincident movement was rated as representing the least friendly atmosphere. Therefore, Hypothesis 1 was accepted and it is assumed that coincidence is a key factor in the inference of a friendly/antagonistic atmosphere.

A significant difference between parallel and forward tilt was found in the incoincidence condition. However, there was no difference between the forward and parallel tilts for coincidence movement. Therefore, Hypothesis 2 was partly rejected. Some participants reported that the forward and incoincident tilt reminded them of “interaction,” such as response or feedback, for example, when a person talks to another and then he or she replies. It is believed that the forward incoincident tilt was suggestive of a relationship friendly enough for responding or providing a feedback. On the contrary, the parallel incoincident tilt did not easily express interaction because this tilt never had a “mutual” orientation, as the shapes always had the same direction. Thus, the differences in terms of perceived interaction between the forward incoincident and the parallel incoincident tilts might cause the significant difference in the friendliness rating.

On the other hand, it can be assumed that not interpreting either of the tilts as an interaction might be the reason for the lack of a significant difference in the friendliness rating between the forward coincident and the parallel coincident tilts. Coincident movement is readily associated with coincident controlled machines, such as a conveyor belt in a factory or the windscreen wipers of a car. It is therefore unlikely for their movement to be interpreted as interaction. The results of Item 2 provided partial evidence for the idea that coincident patterns of movement might not be understood as an interaction and showed that such patterns were interpreted as significantly less biological than incoincident patterns. Coincident movement might have been seen as monotonic and mechanical, and this difficulty to interpret it as interaction might have led to the absence of a significant difference between the forward coincident and the parallel coincident tilts in terms of the friendliness rating.

The results showed that the trend in rating scores inclined towards the middle value (i.e., 2 = *neither*). This trend suggests that participants evaluated the stimuli ambiguously, leading to ineffective answers. To clarify this, we analyzed the deviation of rating scores in each condition from 2. We calculated these deviation values |*x_i_* −2| in each condition (*x_i_* is the rating score of participant *i*) and subjected them to a two-way repeated-measure ANOVA. There was a significant main effect of tilt for friendliness/antagonism, *F*(2, 198) = 7.41, *p* < .01. We therefore conducted multiple comparisons (Bonferroni corrected), which revealed significant differences between the tilts, as follows: parallel = backward < forward (MSE = 0.284, *p* < .05). We also found a main effect of timing for friendliness/antagonism, *F*(1, 99) = 24.89, *p* < .01, and biological/mechanical, *F*(1, 99) = 7.31, *p* < .01, which both revealed a pattern of incoincidence < coincidence. Thus, we obtained significant results even when using the deviation scores from 2 (*neither*), which confirm that participants had effective answers. Moreover, the results indicating strong influences of forward tilt and coincidence on friendliness/antagonism ratings are not inconsistent with the earlier discussion that the coincidence and forward tilt caused the significant differences. Particularly, the fact that forward tilt did not appear to be ambiguous to participants supports the notion that forward tilt is perceived as friendlier compared to backward and parallel tilts. For biological/mechanical ratings, the scores were significantly lower for incoincident movement than for coincident movement, suggesting that the coincident patterns were interpreted of mechanical movement, as mentioned earlier.

Furthermore, there is a possibility that a slight deviation such as in incoincident movement may be interpreted as friendliness, according to studies by Meyer (1956) and Narmour (1990). They reported that deviations from expectations increased emotional response to music (Meyer, 1956; Narmour, 1990; Yuasa, Ohmura, & Katagami, 2014). Similarly, there is a possibility that a slight deviation in movement may convey a positive emotion such as friendliness.

To summarize, our results suggest the possibility that individuals may infer a friendlier atmosphere from coincident movement, and from the combination of incoincidence and forward tilt. The current study suggests that interpreting movements as interaction may be a partly crucial factor to perceive an atmosphere between two objects as friendly. The finding will be useful for designing atmospheres for humans and animated agents/robots, which are novel design methods necessary for creating a better communicative atmosphere for animated agents and robots (Yuasa, 2014).

In addition, we describe the relationship to studies of animacy. This study contributes to animacy studies in elaborating how people discover causality and social responses of objects, and the findings of this study become new examples for future studies on animacy. The current study investigated the expressions that can contribute to a sense of friendliness; these expressions can create the special meaning (friendliness) that a single object could never infer individually. Thus, this study is similar to the animacy studies that make sense by multiple objects, causality and social responses of multiple objects. In the earliest studies, Michotte (1963), Tremoulet and Feldman (2000), and Metelli (1982) investigated causal effects (launching, triggering, etc.) using multiple objects. Spelke, Phillips, and Woodward (1995) also investigated the actions of animated objects and proposed principles to create human-like actions (i.e., social responsiveness). Although these studies are similar to the present study, they still differ because this study created expressions of friendliness using tilt movements without trajectory information. Therefore, the cues of this study are beneficial in that they provide new examples of making sense of multiple objects without trajectories, which may lead to a further our understanding of how people infer causality or social responses.

The cues to creating friendliness in this study can be generalized to communication behaviors in conversations. Yuasa, Mukawa, Kimura, Tokunaga, and Terai (2010) study, which analyzed body postures of the speaker and the listener, proposed the behaviors of animated agents that could comprehend positive or negative attitudes in conversations. The cues from this study can contribute to investigating the behaviors that better communication or create a friendly atmosphere in conversations using tilts and coincidence/incoincidence. These cues can also be useful in analyzing how people understand slightly more complex social behaviors, which must be observed for a certain amount of time, such as following, fighting, and chasing. McAleer and Love (2013) also investigated the comprehension of such behaviors. Since McAleer’s study analyzed only the positions of abstract objects and their changes over time, tilt behaviors and coincidence/incoincidence will be useful as advanced factors to investigate such slightly complex social behaviors.

Moreover, we explain the connections to existing theoretical studies. The “theory of mind” in the field of developmental psychology may be considered a criterion for whether a child can simulate another’s mind (Baron-Cohen & Leslie, 1985; Premack, & Woodruff, 1978; Wimmer & Perner, 1983). It may also be necessary for understanding friendliness/antagonism, because it is difficult to infer social relationships like friendliness/antagonism without simulating others’ minds. In the studies of children playing, Smilansky (1968) and Smilansky and Shefatya (1990) characterized the criteria for sociodramatic play, one of which was “interaction,” which implies that at least two players interact in the context of a play scene. The comprehension of friendliness/antagonism may be needed for the “interaction” criterion in children’s development.

Furthermore, we might discuss the connection between the current study and communication theories (Burgoon, Stern, & Dillman, 1995; Griffin, 1997; Watzlawick, Weakland, & Fisch, 1974). One principle of the interpersonal adaptation theory (Burgoon et al., 1995) is that people biologically and unintentionally behave to achieve “synchronicity” with each other. Coincidental movements that are equivalent to “synchronicity” were considered to be natural human behavior, which might lead to a high evaluation of friendliness. Social interaction theory explains two types of feedback relationships: symmetrical and complementary feedback (Griffin, 1997; Watzlawick et al., 1974). Symmetrical feedback is when one person responds to the other in the same way, while complementary feedback is when participants react in opposite ways. Symmetrical and complementary feedback might be equivalent to parallel and forward tilts. Humans tend to be sensitive to both forms of feedback, which might have resulted in the significant differences in friendliness and biological ratings in our results.

### Limitations

The aim to understand atmosphere perception was achieved using sine functions. However, we must investigate other types of equations in the future; indeed, in our next study, we include other wave phenomena (i.e., beat, resonance, and frequency-locking phenomena). Moreover, in this study, we used the egg shape as a first step to examine communicative atmosphere; however, different results might be obtained if other geometric shapes, like a square or a triangle, are used. Pavlova, Sokolov, and Sokolov (2005) used several types of shapes, such as triangles, ovals, and single lines, in a study on emotional attribution. Thus, other types of shapes need to be used to investigate differences in inferences according to shape. Finally, we did not add any facial features, such as a nose, a mouth, or eyes, to the geometric shapes in our study and used only one level of abstraction for geometric shapes. Kuhlmeier et al. (2004) used geometric shapes such as triangles, squares, and circles with eyes and a nose; Yuasa, Saito, and Mukawa (2009) used a changing level of character abstraction; and Archer (1980), Kimura, Yogo, and Daibo (2005), Kimura, Daibo, and Yogo (2010), and Kito (2004) investigated which particular nonverbal cue was important to infer an interpersonal relationship such as familiarity from photos of individuals. Future studies should try to use different abstraction levels, as well as real images and various kinds of facial and bodily parts. On the other hand, when using less abstraction, the information from superficial elements increases and significant factors might become hidden. Thus, future research should consider which factors are appropriate and execute experiments systematically.

We used only subjective self-rated questionnaires, and an objective evaluation should be used to show rigidity and rigorousness. However, since the current study is the first report to evaluate friendliness, which is expressed by abstract shapes, it is hard to develop an objective evaluation immediately. We think the subjective method is sufficient to evaluate friendliness as a first step and we will be able to find a suitable objective method for future studies (e.g., measurement of reaction times or eye movements).

## Conclusions

In order to investigate how people infer friendly communicative atmosphere from the movements of geometric shapes, we developed animations using several types of tilts and different delays in the movement of two egg shapes. The shapes’ movement was designed based on a sine function to create synchronous reciprocal motion and other types of motion by varying the values. The experimental results showed differences between coincident and incoincident movements in the inference of friendly/antagonistic atmosphere. Moreover, in the case of incoincident movements, the atmosphere was rated as friendlier for forward tilts than for parallel and backward ones. This study suggests that interpreting the movement as an interaction may be an important factor to infer friendliness; individuals may have the capacity to infer a friendly communicative atmosphere from both coincident and incoincident movements interpreted as interaction.
